# Establishment and validation of a predictive nomogram for the risk of premalignant lesions in children with choledochal cyst

**DOI:** 10.3389/fped.2023.1108788

**Published:** 2023-02-03

**Authors:** Ruyue Gao, Meng Ke, Jie Shi, Yandong Zhang, Jizhen Zou, Mei Diao, Long Li

**Affiliations:** ^1^ Department of Pediatric Surgery, Children's Hospital Capital Institute of Pediatrics, Beijing, China; ^2^ Graduate School of Peking Union Medical College, Chinese Academy of Medical Sciences, Beijing, China; ^3^ Department of Pathology, Peking Union Medical College Hospital, Chinese Academy of Medical Sciences and Peking Union Medical College, Beijing, China; ^4^ Department of Pathology, Institute of Basic Medical Sciences, Chinese Academy of Medical Sciences, Peking Union Medical College, Beijing, China; ^5^ Department of Pathology, Children's Hospital Capital Institute of Pediatrics, Beijing, China; ^6^ Department of Pediatric Surgery, Beijing Tsinghua Changgung Hospital, Beijing, China; ^7^ Research Unit of Minimally Invasive Pediatric Surgery on Diagnosis and Treatment (2021RU015), Chinese Academy of Medical Sciences, Beijing, China

**Keywords:** choledochal cyst, pancreatobiliary maljunction, metaplasia, dysplasia, malignancy

## Abstract

**Background:**

Choledochal cyst (CDC) increases the risk (2.5%–30%) of malignancy. Metaplasia and dysplasia have been recognized as premalignant lesions among CDCs. This study aimed to evaluate the risk factors of metaplasia and dysplasia in CDC children.

**Methods:**

Two hundred and ten CDC children who underwent cyst excision and Roux-en-Y hepaticojejunostomy at our institution between July 2020 and November 2021 were included and randomly divided into the training set and validation set. Univariate and multivariate logistic regression analysis were used to identify independent risk factors of premalignant lesions in the training set and build a predictive nomogram. The performance and discriminatory abilities of the nomogram were further assessed and validated in the validation set.

**Results:**

Of the 210 CDC children, 78 (37.1%) patients developed premalignant lesions. Age (OR, 1.011, 95%CI, 1.000–1.022, *P *= 0.046), symptoms duration (OR, 1.021, 95%CI, 1.001–1.042, *P *= 0.036), cyst diameter (OR, 1.737, 95%CI, 1.328–2.273, *P *< 0.001), recurrent attacks of biliary pancreatitis (OR, 3.653, 95%CI, 1.205–11.076, *P *= 0.022), and biliary operation history (OR, 5.860, 95%CI, 1.268–27.084, *P *= 0.024) were identified as independent risk factors. Based on these predictors, a predictive nomogram was generated. The AUC of the nomogram was 0.873 in the training set and 0.793 in the validation set, indicating that it was robust and well calibrated.

**Conclusions:**

A novel nomogram to the individualized risk of premalignant lesions in CDC children was successfully built, on the basis of age, symptoms duration, cyst diameter, recurrent attacks of biliary pancreatitis, and biliary operation history. This nomogram, combined with the final pathological results, can help clinicians to develop more efficient follow-up strategies for the high-risk children with CDC.

## Introduction

Choledochal cyst (CDC) is generally considered a premalignant lesion of cholangiocarcinoma (CCA) (1–3). Malignancies develop in 2.5%–30% of patients with CDC, which is 1,000–2,000 times higher than that in the general population (4–6). Additionally, patients with CDC develop malignancies at a younger age (median 49.5 years) compared with the general CCA population (median 65 years) (1, 7). To date, the youngest CDC patient who progressed to CCA was only 3 years old (8). The exact pathophysiology of this malignant transformation is still unknown, but increasing age is believed to be an important risk factor. It has been reported that the incidence of malignancy has been increasing over the decades, a 0.4% in patients below 18 but nearly 30%–40% for those over 50 years (9–12). In addition, surgical modality and Todani type of CDC have also been suggested to be strongly associated with the malignancy (13–16), which provided the basis for the current treatment concept: complete cyst excision followed by a construction of a biliodigestive anastomosis.

CCA that arises from CDC has an insidious onset and very poor outcome. The majority of CCA patients are usually at an advanced stage, and thus only a few are eligible for surgical resection at the time of diagnosis (17–19). Therefore, early signs of malignancy transformation in CDC patients are crucial for screening and prevention of CCA. In a clinicopathologic study by Katabi and her colleagues, metaplasia was found in 14 of 35 (40%) cysts, 9 of which were dysplasia (64.3%) and 5 were malignant, 3 of the malignant cysts were associated with metaplasia and dysplasia. Furthermore, expression of the p53 and Ki-67 markers increased from metaplasia to dysplasia to carcinoma (20). Other reports have also described hyperplasia, metaplasia and dysplasia in CDC patients, primarily appearing in the para-carcinoma tissues (21–23). The strong correlation between CCA, metaplasia and dysplasia accounts for the “metaplasia-dysplasia-carcinoma” sequence in patients with CDC.

Relative to adult populations, children with CDC are not adequately assessed for risk of subsequent malignancy, a shortcoming attributable to the low incidence of the condition in pediatric populations. In this study, we reevaluated the epithelial changes in these populations, including hyperplasia, metaplasia and dysplasia investigated and independent risk factors for these lesions established. A predictive nomogram to guide clinical decision making in CDC children was also developed in this study.

## Methods

This retrospective study was designed and conducted in accordance with the requirements of the Declaration of Helsinki and the Institutional Review Board of Capital Institute of Pediatrics. Informed consent was received from the parents or legal guardians of CDC patients that participated in this study. Ethical approval from the Ethics Committee of Capital Institute of Pediatrics (Beijing) was also obtained (SHERLL2020038).

### Patients

Two hundred and ten CDC children aged from 1 day to 16 years who underwent cyst excision and Roux-en-Y hepaticojejunostomy at the Capital Institute of Pediatrics, Beijing, China, between July 2020 and November 2021 were included in this study.

### Research methods

Clinical data were collected from patient's medical records in an electronic database, including age, gender, timing of diagnosis (prenatally or otherwise), history of recurrent attacks of biliary pancreatitis, cyst perforation, history of biliary operation, symptoms duration and preoperative serum biochemistry. Cyst size, the presence of pancreatobiliary maljunction (PBM), intraluminal protein plug/calculi and intrahepatic bile duct dilatation revealed by preoperative imaging examinations and intraoperative cholangiography were also noted.

Hematoxylin and eosin-stained sections for study participants were retrieved and reevaluated by three expert pathologists (Jie Shi, Yandong Zhang, and Jizhen Zou). Patient's clinical data was however not disclosed. Degree of inflammation of cyst wall was graded as mild (scattered inflammatory cells limited to the lamina propria), moderate (inflammatory cells extending into the submucosa), and severe (transmural inflammatory cells infiltration). Abnormal epithelial changes including hyperplasia, metaplasia, and dysplasia were documented. An epithelial premalignant lesion was defined as the presence of metaplasia or dysplasia. Dysplasia (biliary intraepithelial neoplasia, abbreviated as BilIN) was graded using a 3-tier grading system (BilIN1-3) according to established criteria (24), corresponding to low-grade, intermediate-grade, and high-grade dysplasia (carcinoma *in situ*), respectively.

### Statistical analysis

IBM SPSS Statistics 23 (IBM, USA) was used to analyze the data. For continuous variables with a normal distribution, the values were expressed as mean ± standard deviations. Variables with a skewed distribution were expressed as the median (interquartile range). Categorical variables were presented as numbers and percentages. Student's t-test, Mann-Whitney U test, chi-squared test, and Fisher's exact test were used for statistical inference. The threshold for statistical significance was set at *P *< 0.05.

The cohort of study participants was randomly assigned to a training set and validation set in a ratio of 2:1 by SPSS software. Variables in the training set that attained *P* values <0.05 in the univariate regression analysis, were subjected to forward selection. The selected variables formed the final binary logistic regression model and were used as predictors of epithelial premalignant lesions. The fit of the model was assessed using the Hosmer–Lemeshow test. The nomogram function in the rms package was employed to generate a nomogram for the aforementioned regression model. A receiver operating characteristic (ROC) curve was computed by the “pROC” package, enabling the investigators to evaluate the predictive ability and calibration of the nomogram. An AUC of the ROC curve (area under the curve) that is >0.75 indicated that the model has a good prediction ability and is well calibrated. Specificity and sensitivity were derived from the curve, the Youden index generated and an optimal cut-off point identified. The nomogram was composed of risk factors, the individual score of risk factors, the total score, and the event risk of epithelial premalignant lesions. The scale represents the range of the available values of the risk factor. The length of the line segment reflects the contribution of the risk factor to the final event. The single score at the top of the chart represents the corresponding risk factor score for different values. The scores for all risk factors were summed to obtain the total score. At this point, the corresponding linear predictive values were identifiable. The bottom graph line represents the prediction probability of epithelial premalignant lesions risk in pathology.

## Results

### Population characteristics

**Figure 1:**
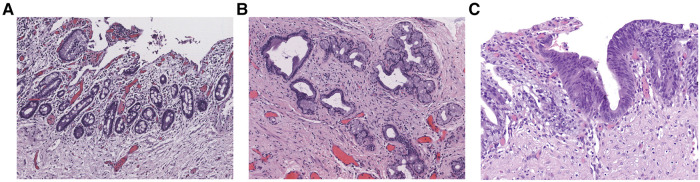
Epithelial premalignant lesions. (**A**) Intestinal metaplasia (Magnification, x100). (**B**) Pyloric gland metaplasia (Magnification, x100). (**C**) Low-grade dysplasia (Magnification, x200).

The clinicopathologic characteristics of the 210 CDC patients enrolled in this study are summarized in Table 1. The median age was 42.5 months (range, 0.03–218 months). 19 (9.0%) patients had previously undergone biliary tract surgery in other hospitals, 7 (36.8%) of whom received endoscopic retrograde cholangiopancreatography (ERCP), endoscopic sphincterotomy (EST), endoscopic retrograde biliary drainage (ERBD) and endoscopic retrograde pancreatic drainage (ERPD) with stents. 5 (26.3%) of the 19 patients underwent cholecystectomy, choledochotomy and T tube drainage, 3 (15.8%) received cystojejunostomy, 2 (10.5%) bile duct irrigation and cholecystostomy, 1 (5.3%) percutaneous transhepatic biliary drainage (PTBD) and cholecystojejunostomy, and 1 (5.3%) cholecystectomy, ERCP, EST and ERPD with stents. Based on the pathological results, 78 (37.1%) patients were confirmed to have premalignant lesions (Figure 1), 42 (53.9%) of whom presented with intestinal metaplasia, 10 (12.8%) with pyloric gland metaplasia, 14 (17.9%) with both intestinal metaplasia and pyloric gland metaplasia, and 12 (15.4%) with dysplasia (10 with concurrent metaplasia). All the identified dysplasia were classified as low-grade as per the BilIN1-3 criteria. Two hundred and ten patients were divided into the training set (*n* = 140) and the validation set (*n* = 70) by SPSS software. The incidence of premalignant lesions was 36.4% and 38.6% in the training and validation sets, respectively. There were no statistically significant differences in baseline characteristics between the two sets (*P *> 0.05, Table 1).

**Table 1:** Baseline characteristics (*N* = 210).

Characteristics	All cohort, *n* = 210	Training set, *n* = 140	Validation set, *n* = 70	*P* value
Age (month), M(IQR)	42.5 (4.4, 103.0)	41.5 (4.3, 96.8)	43.0 (5.4, 109.3)	0.803
Gender				0.383
Male	58 (27.6%)	36 (25.7%)	22 (31.4%)	
Female	152 (72.4%)	104 (74.3%)	48 (68.6%)	
Prenatal diagnosis				0.672
Yes	64 (30.5%)	44 (31.4%)	20 (28.6%)	
No	146 (69.5%)	96 (68.6%)	50 (71.4%)	
Recurrent attacks of biliary pancreatitis				0.168
Yes	64 (30.5%)	47 (33.6%)	17 (24.3%)	
No	146 (69.5%)	93 (66.4%)	53 (75.7%)	
Symptom duration (month), M(IQR)	6.0 (2.0, 24.0)	7.0 (3.0, 24.0)	5.0 (1.0, 25.5)	0.300
PBM				0.051
Yes	160 (76.2%)	101 (72.1%)	59 (84.3%)	
No	50 (23.8%)	39 (27.9%)	11 (15.7%)	
Protein plug/Calculi				0.433
Yes	95 (45.2%)	66 (47.1%)	29 (41.4%)	
No	115 (54.8%)	74 (52.9%)	41 (58.6%)	
Diameter (cm), M(IQR)	2.5 (2.0, 4.1)	2.5 (1.8, 4.4)	2.9 (2.0, 4.1)	0.574
Length (cm), M(IQR)	6.0 (4.0, 7.2)	6.0 (4.0, 7.2)	6.0 (4.0, 7.2)	0.893
Perforation				0.357
Yes	24 (11.4%)	18 (12.9%)	6 (8.6%)	
No	186 (88.6%)	122 (87.1%)	64 (91.4%)	
Intrahepatic bile duct dilatation				0.768
Yes	93 (44.3%)	61 (43.6%)	32 (45.7%)	
No	117 (55.7%)	79 (56.4%)	38 (54.3%)	
Biliary operation history				0.234
Yes	19 (9.0%)	15 (10.7%)	4 (5.7%)	
No	191 (91.0%)	125 (89.3%)	66 (94.3%)	
ALT (U/L), M(IQR)	27.1 (14.0, 73.0)	29.0 (14.5, 79.0)	22.8 (12.5, 62.1)	0.415
AST (U/L), M(IQR)	34.8 (25.4, 60.5)	36.0 (25.4, 64.7)	33.3 (25.5, 53.3)	0.450
ALP (U/L), M(IQR)	243.0 (179.0, 318.5)	244.0 (175.0, 322.5)	243.0 (181.0, 313.0)	0.678
GGT (U/L), M(IQR)	103.4 (29.0, 285.0)	106.0 (33.6, 329.5)	79.0 (21.7, 207.0)	0.098
TBIL (umol/L), M(IQR)	12.3 (7.8, 38.4)	12.7 (8.0, 44.7)	11.2 (7.5, 34.9)	0.421
DBIL (umol/L), M(IQR)	3.7 (2.1, 10.1)	3.8 (2.2, 11.4)	2.0 (1.1, 3.0)	0.190
AMY (U/L), M(IQR)	57.0 (15.0, 93.0)	58.0 (15.0, 94.5)	50.0 (15.0, 92.3)	0.767
Cholangitis				0.837
Mild	139 (66.2%)	92 (65.7%)	47 (67.1%)	
Moderate-severe	71 (33.8%)	48 (34.3%)	23 (32.9%)	
Premalignant lesions^a^				0.762
Yes	78 (37.1%)	51 (36.4%)	27 (38.6%)	
No	132 (62.9%)	89 (63.6%)	43 (61.4%)	

*NOTE:* The results are presented as median (interquartile range) for continuous variables, and number (percentage) for categorical variables. Abbreviations: M(IQR), median (quartile range); PBM, pancreaticobiliary maljunction; ALT, alanine aminotransferase; AST, aspartate transaminase; ALP, alkaline phosphatase; GGT, *γ*-glutamyl transferase; TBIL, total bilirubin; DBIL, direct bilirubin; AMY, amylase.

^a^ Premalignant lesion was defined by the presence of metaplasia or dysplasia.

### Evaluation of risk factors for epithelial premalignant lesions in the training set

In the univariate regression analysis, older age (*P *< 0.001), non-prenatal diagnosis (*P *= 0.003), a history of recurrent attacks of biliary pancreatitis (*P *= 0.004), longer symptoms duration (*P* < 0.001), greater cyst diameter (*P *= 0.001) and length (*P *< 0.001), a history of biliary tract surgery (*P *= 0.001), and moderate to severe cholangitis (*P *= 0.043) were significantly associated with a higher likelihood of premalignant lesions (Table 2). Variables with *P* values <0.05 in the univariable regression analysis were entered into a forward stepwise variable selection procedure to generate a final binary logistic regression model consisting of predictors with *P* values < 0.05.

**Table 2:** Univariate and multivariate logistic regression analysis in the training set.

Variables	Univariate logistic regression					Multivariate logistic regression				
	B	S.E.	Wald	*P* value	OR (95% CI)	B	S.E.	Wald	*P* value	OR (95% CI)
Age	0.020	0.004	25.860	<0.001	1.020 (1.012–1.028)	0.011	0.005	3.999	0.046	1.011 (1.000–1.022)
Prenatal diagnosis	−1.295	0.441	8.604	0.003	0.274 (0.115–0.651)					
Recurrent attacks of biliary pancreatitis	1.074	0.373	8.315	0.004	2.928 (1.411–6.078)	1.295	0.566	5.239	0.022	3.653 (1.205–11.076)
Symptom duration	0.034	0.009	15.426	<0.001	1.035 (1.017–1.052)	0.021	0.010	4.374	0.036	1.021 (1.001–1.042)
Diameter	0.314	0.093	11.413	0.001	1.369 (1.141–1.643)	0.552	0.137	16.236	<0.001	1.737 (1.328–2.273)
Length	0.356	0.087	16.865	<0.001	1.427 (1.204–1.691)					
Biliary operation history	2.177	0.674	10.441	0.001	8.821 (2.355–33.036)	1.768	0.781	5.125	0.024	5.860 (1.268–27.084)
Cholangitis (Moderate-severe)	0.743	0.367	4.098	0.043	2.103 (1.024–4.319)					
Constant						−4.200	0.708	35.164	<0.001	0.015 (-)

Abbreviations: B, *β* coefficient; SE, standard error; Wald, *χ*2 value of Wald test; OR, odds ratio.

**Figure 2:**
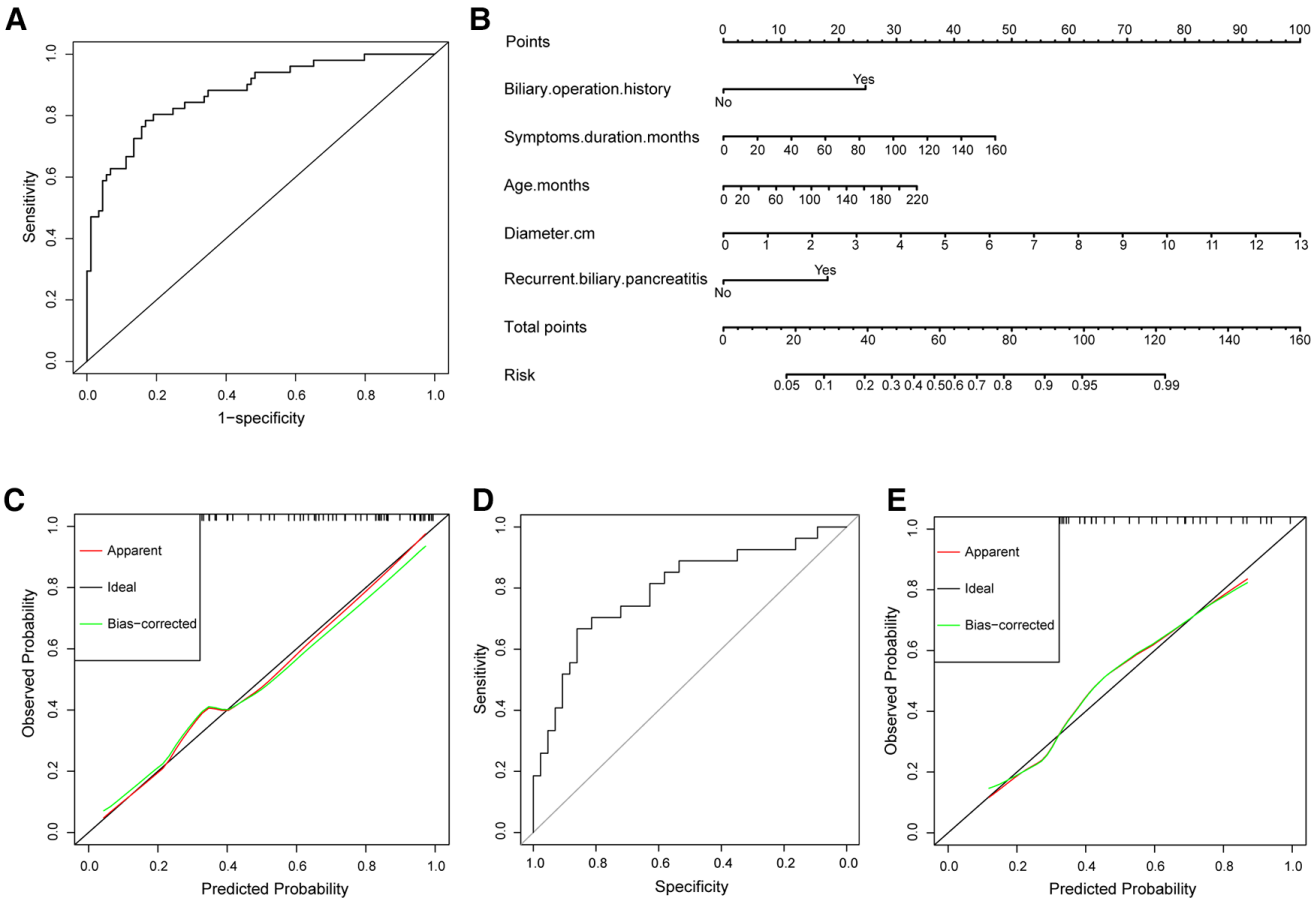
(**A**) The ROC curve of the regression model in the training set. The area under the curve was 0.873 (95%CI, 0.812–0.934). (**B**) Nomogram for premalignant lesions risk. (**C**) Calibration of the nomogram to predict the probability of premalignant lesions in the training set. (**D**) The ROC curve of the regression model in the validation set. The area under the curve was 0.793 (95% CI, 0.680–0.907). (**E**) Calibration of the nomogram to predict the probability of premalignant lesions in the validation set.

In the multivariate regression analysis, with results reported as odds ratio (95%CI), age (OR, 1.011, 95%CI, 1.000–1.022, *P *= 0.046), symptoms duration (OR, 1.021, 95%CI, 1.001–1.042, *P *= 0.036), cyst diameter (OR, 1.737, 95%CI, 1.328–2.273, *P *< 0.001), recurrent attacks of biliary pancreatitis (OR, 3.653, 95%CI, 1.205–11.076, *P *= 0.022), and biliary operation history (OR, 5.860, 95%CI, 1.268–27.084, *P *= 0.024) were independently associated with premalignant lesions (Table 2). The goodness-of-fit test value of the Hosmer–Lemeshow (HL) was 0.590, higher than 0.05, indicating that the regression model fit the recorded data well. The ROC curve of the regression model for predicting the occurrence of premalignant lesions is shown in Figure 2A. The AUC was 0.873 (95%CI, 0.812–0.934), indicating that the model had good predictive ability and was well-calibrated. The optimal cutoff value of the model was 0.3121468, the Youden index was 0.615, the sensitivity was 78.4%, and the specificity was 83.1%.

### Development and validation of the nomogram for epithelial premalignant lesions risk in the training set

The independently associated risk factors were used to form a premalignant lesions risk estimation nomogram (Figure 2B). The nomograhm demonstrated good accuracy in estimating the risk of premalignant lesions. The calibration plot graphically showed good comparability between the risk estimates computed by the nomogram and histopathologic confirmation from surgical specimens (Figure 2C).

### Validation of the nomogram in the validation set

The ROC curve of the regression model for the validation set is shown in Figure 2D. The predictive capability of the model was robust, evidenced by the AUC of 0.793 (95% CI, 0.680–0.907). At a cutoff value of 0.3121468, the sensitivity and specificity were 66.7% and 81.4%, respectively. The calibration curve of the nomogram showed good agreement (Figure 2E), demonstrating the good discriminatory ability of the nomogram.

## Discussion

Malignant transformation is a significant concern in patients with CDC. The completion of the surgery does not mean the end of the treatment, precision follow-up is especially necessary to improve the long-term prognosis of CDC. Currently, studies on the risk factors of malignant transformation in CDC children are lacking due to the extremely low incidence. However, early identification of high-risk individuals and the quantitative analysis of the risk are vital steps to achieve precision follow-up and, as a result, to screen and prevent malignancy. Therefore, this was explored in this study.

Metaplastic lesions are considered to be a premalignant development among patients with CDC (20–22). Metaplasia, including intestinal and pyloric gland metaplasia, and dysplasia accounted for 37.1% (78/210) of cases in this study. Five independent risk factors for these lesions were identified, that are age, symptoms duration, cyst diameter, recurrent attacks of biliary pancreatitis and a history of biliary tract surgery.

Malignant transformation generally requires a prolonged exposure to carcinogenic factors. Thus, advanced age is an acknowledged risk factor (4, 25). In this study, the risk of epithelial premalignant lesions in CDC patients similarly increased with age, consistent with the observations by Komi et al. (21). This suggested that the process is one that escalates from metaplasia to neoplasia, the completion of malignant transformation usually requiring several decades. Cholestasis within cysts has been proposed as a key precipitant of the development of metaplasia and malignancy (26), the secondary bacterial overgrowth being the main trigger for the transformation of epithelium within cysts. Therefore, early operation may impede this process and hence decrease the malignant potential. In a fashion similar to age, both symptoms duration and cyst diameter are also time-related factors that present increased risk.

In addition to time-related factors, a history of biliary tract surgery before cyst excision increased the risk of epithelial metaplasia and dysplasia. Surgical treatments for CDC have evolved significantly over the past decades (27–31). The earlier drainage procedures have been long abandoned due to the high rate of malignancies that ensue, especially when internal drainage operations are used. It has been reported that patients who have had previous internal drainage operations developed malignancies at relatively young ages (32, 33), a circumstance in which enterokinase was implicated. It has been postulated that the enterokinase enhances the activation of refluxed pancreatic enzymes, consequently accelerating the destruction of biliary epithelium (34). Currently, complete cyst excision followed by a construction of a biliodigestive anastomosis is established as a standard surgical procedure. Nevertheless, ERCP procedures prior to complete cyst excision have been well implemented in CDC children using advanced endoscopes and modern accessories (35–37). A retrospective review by Makita and his colleagues revealed that diagnostic and therapeutic ERCP including ERBD, ERPD, and EST is safe and effective in removal of protein plug and symptomatic relief of CDC (36, 37). In our study, 8 (3.8%) CDC patients underwent ERCP procedures before complete cyst excision, the ERCP-cyst excision interval ranged from 1 to 36 months. Although symptoms were subsequently relieved in these children, 7 (87.5%) developed premalignant lesions. The causal relationship between ERCP procedures and premalignant lesions is likely due to the deferral of radical surgery, and potentially also due to the long-term stimulation and inflammatory responses induced by stents. Furthermore, owing to the extensive adhesions and inflammatory bleeding that ensues from the surgery, cyst excision is considerably difficult in children who have had ERCP procedures, and for these patients, the duration of surgery can be quite prolonged. As a consequence, although ERCP procedures could alleviate the symptoms and have certain diagnostic value for CDC, radical surgery should be performed as soon as possible.

PBM is a common anatomic anomaly in patients with CDC and predisposes them to biliary cancer and pancreatitis (38, 39). Persistent reflux of pancreatic juice into the bile duct is thought to be involved in malignant transformation. It has been reported that refluxed proteolytic pancreatic enzymes and phospholipase A2 are activated in the bile duct, stimulating the production of highly cytotoxic substances such as lysolecithin. These result in chronic inflammation, repeated cycles of damage and healing of the biliary epithelium and promote malignant transformation (40). In our study, an overwhelming 76.2% of the CDC patients had concomitant PBM. However, the recurrent biliary pancreatitis, not PBM, was identified as an independent risk factor for premalignant lesions, which is attributed to the distal stenosis of the choledochus, another typical feature of CDC (39, 41). Among CDC patients with this stenosis, the increased intraluminal pressure prevented PBM-associated bidirectional bile/pancreatic juice reflux and pancreatitis, protecting the biliary epithelium as a result. On the other hand, for patients characterized by recurrent biliary pancreatitis, early diagnosis of CDC is difficult due to the milder biliary dilatation, clinicians should be vigilant about this atypical presentation, and diagnostic ERCP or intraoperative cholangiography is recommended to obtain a definite diagnosis.

A small subset of patients develops CCA after cyst excision. In a study by Shigeru Ono and his colleagues, a 26-year-old male was reported to have developed CCA after cyst excision at the age of 5 months (42). An increased risk of malignant transformation was described by Kobayashi et al., who reported on three cases of CCA at 2, 8, and 19 years after cyst excision (43). The reported incidences of postoperative malignancy were 1.6% at 15 years, 3.9% at 20 years, and 11.3% at 25 years (44), indicating that surgical treatment for CDC could not eliminate the risk of malignant development. Subsequent malignancy most commonly occurs at the hepatic duct or near the bilioenteric anastomosis, followed by the intrahepatic, and intrapancreatic bile ducts (44–46). Current understanding predicates that the malignancy may be secondary to the chronic inflammation that emerges after bilioenteric anastomosis. Nevertheless, any portion of the biliary tree in addition to the dilated bile duct is never completely normal. Therefore, regular follow-up is imperative, especially in patients who developed metaplasia and dysplasia in the resected cyst specimen given the “field effect” along the intrahepatic and extrahepatic biliary tree.

Scholars globally recommend life-long follow-up for patients with CDC, at least once annually. Sanjani et al. introduced a follow-up protocol that children and adolescents should be seen yearly after cyst excision, serum biochemistry and abdominal ultrasound examination being key at these review visits. In adults, CA19-9 levels should be monitored annually to ensure that malignancy does not go undetected (6). However, in specialist pediatric hospitals, patient compliance with life-long follow-up remains poor specifically when adulthood is reached. As such, we have some recommendations. Firstly, pathologists should be informed of the critical clinical information pertaining to the patient. This ensures that the pathologist and surgeon are cognizant of the degree of risk of epithelial premalignant lesions. Secondly, observed metaplasia and dysplasia ought to also be documented in detail in pathology reports, to remind the care team and patients of malignancy risk. This may make long-term follow-up easier and more effective. Thirdly, the entire cyst should be sampled totally for microscopic examination, ensuring that focal lesions do not elude the pathologist. Last but not least, for patients with normal epithelium, if such risk factors exist, active and frequent follow-up is also essential.

There are some limitations to this study. First, this study was based on a retrospective cohort in which pathological sections which may only be a portion of the entire cyst were retrieved from the pathology archives, and thus we cannot ascertain whether the pathological characteristics were representative of the entire cyst. The incidence of epithelial premalignant lesions might be underestimated since these lesions are often focal. As a result, during pathologic examination, the entire cyst should be examined to determine the future risk of malignancy, as we have already mentioned above. Second, whether metaplasia and dysplasia are early signs of malignancy need to be clarified in studies with longer follow-up, and we will report this longitudinal data in our future investigations. Third, this was a single-center cohort and thus lacked external validation. We hope these limitations can be resolved in the future.

Overall, the present study supports that metaplasia and dysplasia are premalignant lesions in patients with CDC. Although more current data involving longitudinal follow-ups is lacking, preventing such lesions and close follow-up is central to the prevention and early detection of malignant transformation. Further molecular biological investigations are, however, required to elucidate the molecular changes across the histologic spectrum ranging from normal to metaplasia to neoplasia. Furthermore, the predictive nomogram established using factors available to the surgeon is intuitive and individualized to help screen high-risk individuals and alert surgeons and pathologists to give extra attention and conduct more detailed pathological examinations.

## Conclusion

A robust nomogram developed in this study revealed that age, symptoms duration, cyst diameter, recurrent attacks of biliary pancreatitis, and biliary operation history are independent risk factors for epithelial premalignant lesions in children with CDC. Once the CDC has been diagnosed, radical surgery should be performed as soon as possible to prevent premalignant and potentially even malignant lesions. This predictive nomogram, combined with the final pathological results, can help clinicians to develop more efficient follow-up strategies for the high-risk children with CDC.

## Data Availability

The original contributions presented in the study are included in the article/Supplementary Material, further inquiries can be directed to the corresponding author/s.
